# Using clinical parameters to predict prostate cancer and reduce the unnecessary biopsy among patients with PSA in the gray zone

**DOI:** 10.1038/s41598-020-62015-w

**Published:** 2020-03-20

**Authors:** Junxiao Liu, Biao Dong, Wugong Qu, Jiange Wang, Yue Xu, Shuanbao Yu, Xuepei Zhang

**Affiliations:** 1 Department of Urology, The First Affiliated Hospital of Zhengzhou University, Zhengzhou, China; 2 Department of Pathology, The First Affiliated Hospital of Zhengzhou University, Zhengzhou, China; 3 Key Laboratory of Precision Diagnosis and Treatment for Chronic Kidney Disease in Henan Province, Zhengzhou, China

**Keywords:** Cancer, Urology

## Abstract

The gold standard for prostate cancer (PCa) diagnosis is prostate biopsy. However, it remines controversial as an invasive mean for patients with PSA levels in the gray zone (4–10 ng/mL). This study aimed to develop strategy to reduce the unnecessary prostate biopsy. We retrospectively identified 235 patients with serum total PSA testing in the gray zone before prostate biopsy between 2014 and 2018. Age, PSA derivates, prostate volume and multiparametric magnetic imaging (mpMRI) examination were assessed as predictors for PCa and clinically significant PCa with Gleason score ≥ 7 (CSPCa). Univariate analysis showed that prostate volume, PSAD, and mpMRI examination were significant predictors of PCa and CSPCa (*P* < 0.05). The differences of diagnostic accuracy between mpMRI examination (AUC = 0.69) and other clinical parameters in diagnostic accuracy for PCa were not statistically significant. However, mpMRI examination (AUC = 0.79) outperformed prostate volume and PSAD in diagnosis of CSPCa. The multivariate models (AUC = 0.79 and 0.84 for PCa and CSPCa) performed significantly better than mpMRI examination for detection of PCa (*P* = 0.003) and CSPCa (*P* = 0.036) among patients with PSA level in the gray zone. At the same level of sensitivity as the mpMRI examination to diagnose PCa, applying the multivariate models could reduce the number of biopsies by 5% compared with mpMRI examination. Overall, our results supported the view that the multivariate model could reduce unnecessary biopsies without compromising the ability to diagnose PCa and CSPCa. Further prospective validation is required.

## Introduction

Prostate cancer (PCa) is the most commonly diagnosed cancer and second leading cause of cancer death in Western countries^1^. Although the incidence of PCa in China is lower than that in Western countries, PCa has become a serious threat to the health of men due to the aging population, changing diets, and availability of physical examination or medical screening in China^2,3^. The gold standard for PCa diagnosis is prostate biopsy. However, Prostate biopsy is an invasive procedure that can come with physical and psychological distress, and is controversial for men with PSA levels in the gray zone^4^.

Localized PCa usually does not present with symptoms, the selection of men for qualifying for prostate biopsy mainly rely on serum Prostate-specific antibodies (PSA) derivates [total PSA (tPSA), free PSA (fPSA), PSA density (PSAD), free/total (f/t)PSA, (f/t)/PSAD], and mpMRI^5,6^. PSA are widely used as an initial screening test for PCa. However, the specificity of total PSA (tPSA) is low when the serum tPSA level is in the gray zone (4–10 ng/ml)^7^. The free/total (f/t)PSA may be adversely affected by several pre-analytical and clinical factors (e.g., instability of fPSA, and variable assay characteristics)^8,9^. A systematic review found the pooled sensitivity of fPSA is 70% in men with a tPSA of 4–10 ng/ml^10^. Porcaro at al. showed a negative association between PCa and prostate volume^11^. Some studies have validated the clinical utility of mpMRI for the detection of clinically significant prostate cancer with Gleason score ≥ 7 (CSPCa) and to guide clinical decisions regarding biopsy^12,13^. Some indolent PCa could be dynamically monitored and do not necessarily require active treatment. The major challenge is to improve the detection of CSPCa or high-grade PCa at early stage^14^.

As far as we know, the knowledge about the performance of PSA derivates, prostate volume, and mpMRI examination in detecting of PCa and CSPCa in men with PSA level in the gray zone is limited. In our study, we evaluated the diagnostic accuracy of age, tPSA, fPSA, (f/t)PSA, PSAD, prostate volume and mpMRI examination for predicting PCa and CSPCa, respectively. Additionally, multivariate models to predict PCa and CSPCa were developed among cases with tPSA level in the gray zone. This study will be helpful for establishing the clinical parameter-based diagnostic model of PCa and CSPCa among Chinese population, thereby reducing unnecessary prostate biopsy, avoiding overtreatment, and selecting the best clinical strategy.

## Results

A total of 235 patients with tPSA level in the gray zone were included in this study. All patients obtained a clear pathological diagnosis. Prostate biopsy results were negative for 179 (76.2%) patients (non-PCa group) and positive for 56 (23.8%) patients (PCa group). Of the PCa cases, 28 were with Gleason score ≤ 6, and 28 were with Gleason score ≥ 3 + 4 (Table 1).

**Table 1:** Characteristics of clinical parameters for cases by pathological results with PSA level in the gray zone.

Parameter	Overall				Prostate cancers		
	Total	PCa (n = 56)*	Non-PCa (n = 179)*	*P*^a^	CSPCa (n = 28)*	Non-CSPCa (n = 28)*	*P*^b^
Age (yrs)	66 (60–72)	70 (61–75)	65 (60–70)	0.052	66 (60–75)	71 (63–75)	0.231
tPSA (ng/ml)	7.3 (5.7–8.5)	7.5 (5.6–8.4)	7.2 (5.7–8.6)	0.824	7.8 (6.3–8.9)	6.7 (5.5–7.9)	0.225
fPSA (ng/ml)	1.02 (0.66–1.36)	0.97 (0.69–1.23)	1.04 (0.66–1.40)	0.633	0.97 (0.67–1.23)	0.96 (0.74–1.28)	0.935
PV (ml)	49 (33–71)	31 (26–50)	52 (37–73)	<0.001	32 (26–49)	30 (24–64)	1.000
(f/t)PSA	0.15 (0.10–0.21)	0.15 (0.11–0.19)	0.15 (0.10–0.21)	0.764	0.13 (0.11–0.22)	015 (0.11–0.18)	0.818
PSAD (ng/ml^2^)	0.07 (0.05–0.11)	0.10 (0.07–0.15)	0.07 (0.05–0.10)	<0.001	0.09 (0.08–0.15)	0.10 (0.06–0.15)	0.780
mpMRI, No. (%)				<0.001			0.017
Total	210 (100)	50 (100)	160 (100)		24 (100)	26 (100)	
Suspicious	40 (19)	19 (38)	21 (13)		13 (54)	6 (23)	
Equivocal	37 (18)	13 (26)	24 (15)		7 (29)	6 (23)	
Negative	133 (63)	18 (36)	115 (72)		4 (17)	14 (54)	

*Data are presented as median (interquartile range) unless other indicated. Denominators for testing of fewer cases than full group are indicated.

^a^The *P* values are comparisons between PCa and non-PCa group.

^b^The *P* values are comparisons between CSPCa and non-CSPCa group.

PSA: prostate-specific antigen; tPSA: total PSA; fPSA: free PSA; PV: prostate volume; f/tPSA: free PSA/total PSA; PSAD: PSA density; mpMRI: multiparametric magnetic resonance imaging; PCa: prostate cancers; non-PCa: non-prostate cancers; CSPCa: clinically significant prostate cancers; non-CSPCa: non-clinically significant prostate cancers.

### Characteristics of clinical parameters for patients by pathological results

The median age was 66 years (interquartile range, IQR: 60–72). And the median tPSA, fPSA, and (f/t)PSA were 7.3 ng/ml (IQR: 5.7–8.5), 1.02 ng/ml (IQR: 0.66–1.36), and 0.15 (IQR: 0.10–0.21), respectively. The PCa and non-PCa groups did not differ significantly with regard to age (*P* = 0.052), tPSA (*P* = 0.824), fPSA (*P* = 0.633), and (f/t)PSA (*P* = 0.764) (Table 1). The median of prostate volume was 49 ml (IQR: 33–71). The prostate volume of the PCa group was smaller than that of the non-PCa group (*P* < 0.001). Conversely, the PSAD of the PCa group were higher than that of the Non-PCa group (*P* < 0.001) (Table 1). Of the 235 cases, 210 performed mpMRI examination. The number of suspicious, equivocal, and negative for presence of PCa were 40 (19%), 37 (18%), and 133 (63%) based on the mpMRI reports, respectively (Table 1). The distributions of mpMRI results were significantly different between PCa and non-PCa groups (*P* < 0.001). Additionally, the differences for the mpMRI results were significant between CSPCa and non-CSPCa group (*P* = 0.017). The differences for other clinical parameters were not significant between CSPCa and non-CSPCa group (Table 1).

### Univariate analysis of risk factors for PCa and CSPCa

In univariate analysis, the risk of PCa increased with age (OR = 1.04, *P* = 0.025), log-transformed PSAD (OR = 12.81, *P* < 0.001), and grade of mpMRI examination, but was inversely associated with prostate volume (OR = 0.98, *P* = 0.004) (Table 2). The diagnostic accuracy of mpMRI examination (AUC = 0.69) was similar with other single parameters: age (AUC = 0.59, *P* = 0.089), prostate volume (AUC = 0.68, *P* = 0.881), and PSAD (AUC = 0.67, *P* = 0.724) in prediction of PCa. The prostate volume (OR = 0.98, *P* = 0.028), log-transformed PSAD (OR = 2.82, *P* = 0.004), and mpMRI examination (*P* < 0.001) were significant predictors of CSPCa (Table 3). The mpMRI examination (AUC = 0.79) outperformed prostate volume (AUC = 0.69) and PSAD (AUC = 0.68) in diagnostic of CSPCa. The best mpMRI cut-off value was “suspicious” of PCa for predicting of CSPCa, which provided sensitivity of 0.833, specificity of 0.694.

**Table 2:** Uni- and multivariate logistic regression analysis for prediction of prostate cancers.

Parameter	Univariate analysis		Multivariate analysis		
	OR (95 CI)	*P*	Coefficient	OR (95% CI)	*P*
Intercept	NA	NA	3.929	NA	0.157
Age (yrs)	1.04 (1.00–1.08)	0.025	0.051	1.05 (1.01–1.10)	0.017
tPSA (ng/ml)	1.03 (0.87–1.23)	0.723	−0.432	0.65 (0.46–0.91)	0.012
fPSA (ng/ml)	0.96 (0.61–1.52)	0.874	NA	NA	NA
PV (ml)	0.98 (0.97–0.99)	0.004	0.035	1.04 (1.01–1.07)	<0.001
(f/t)PSA	0.31 (0.01–9.15)	0.497	NA	NA	NA
PSAD (ng/ml^2^)	2.81 (1.62–4.85)*	<0.001	3.184*	24.2 (4.17–140)*	<0.001
mpMRI1	7.52 (2.07–27.4)	0.002	1.560	4.76 (1.85–12.2)	0.001
mpMRI2	15.5 (4.70–51.3)	<0.001	1.823	6.19 (2.56–15.0)	<0.001

PSA: prostate-specific antigen; tPSA: total PSA; fPSA: free PSA; PV: prostate volume; (f/t)PSA: free PSA/total PSA;

PSAD: PSA density; mpMRI: multiparametric magnetic resonance imaging; OR: odds ratio; CI: confidence interval;

NA: not applicable; *Parameter was log-transformed; mpMRI1: equivocal VS negative; mpMRI2: suspicious VS negative.

**Table 3:** Uni- and multivariate logistic regression analysis for prediction of clinically significant prostate cancers.

Parameter	Univariate analysis		Multivariate analysis		
	OR (95 CI)	*P*	Coefficient	OR (95% CI)	*P*
Intercept	NA	NA	−0.805	NA	0.455
Age (yrs)	1.01 (0.97–1.06)	0.514	NA	NA	NA
tPSA (ng/ml)	1.15 (0.91–1.45)	0.237	NA	NA	NA
fPSA (ng/ml)	1.08 (0.61–1.93)	0.789	NA	NA	NA
PV (ml)	0.98 (0.96–1.00)	0.028	NA	NA	NA
(f/t)PSA	0.40 (0.00–34.5)	0.687	NA	NA	NA
PSAD (ng/ml^2^)	2.82 (1.41–5.60)*	0.003	1.113*	3.04 (1.32–7.04)*	0.009
mpMRI1	7.52 (2.07–27.4)	0.002	2.261	9.60 (2.52–36.6)	<0.001
mpMRI2	15.5 (4.70–51.3)	<0.001	2.709	15.0 (4.42–51.0)	<0.001

PSA: prostate-specific antigen; tPSA: total PSA; fPSA: free PSA; PV: prostate volume; (f/t)PSA: free PSA/total PSA;

PSAD: PSA density; mpMRI: multiparametric magnetic resonance imaging; OR: odds ratio; CI: confidence interval;

NA: not applicable. *Parameter was log-transformed; mpMRI1: equivocal VS negative; mpMRI2: suspicious VS negative.

### Multivariate analysis of risk factors for PCa and CSPCa

**Figure 1:**
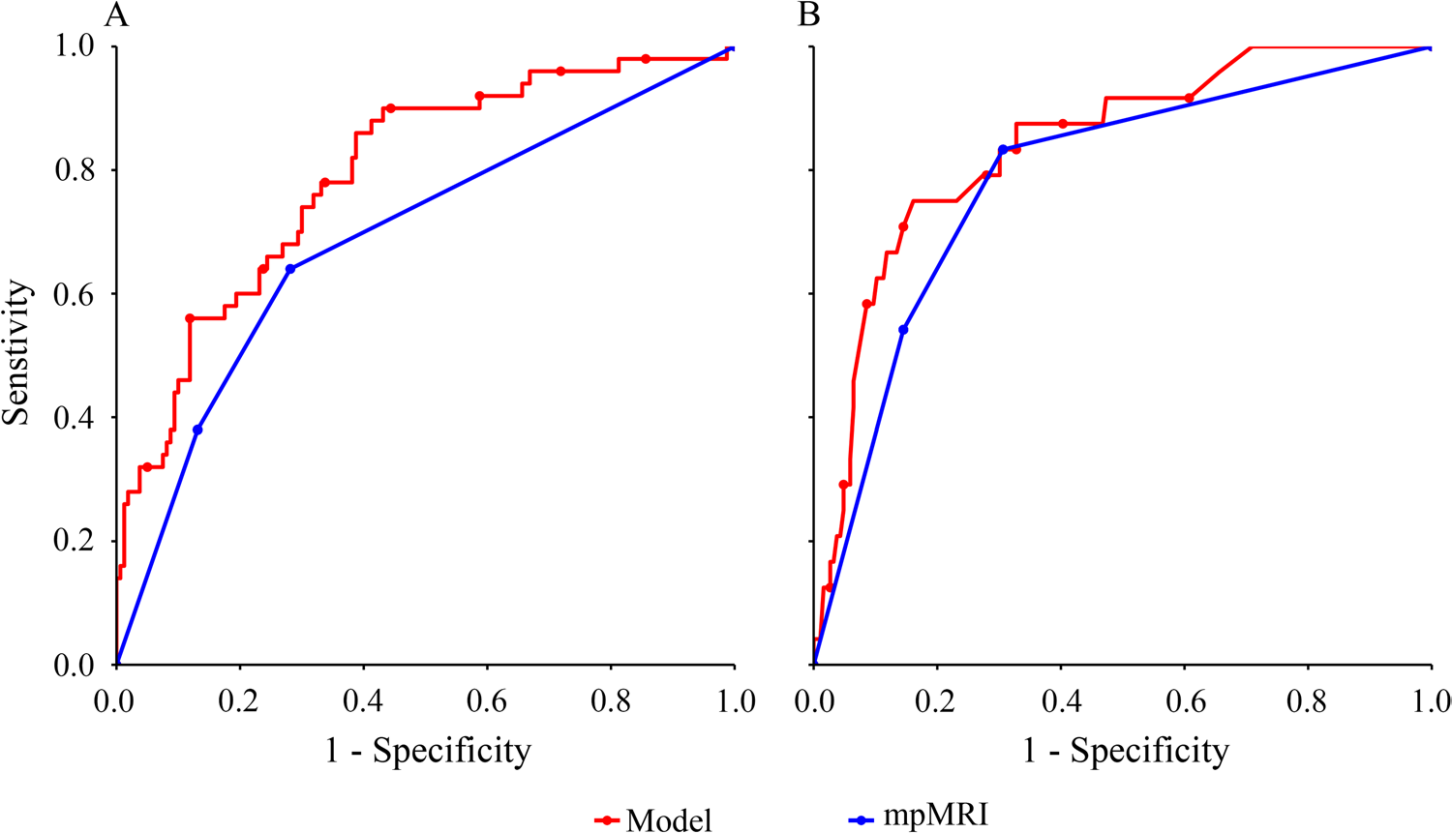
The comparison between mpMRI examination and the multivariate model in diagnostic efficacy.

In a stepwise AUC analysis, age (*P* = 0.017), tPSA (*P* = 0.012), PSAD (*P* < 0.001), and mpMRI examination (*P* < 0.001) reminded in the model for detection of PCa (Table 2). The multivariate model for CSPCa was established including PSAD (*P* = 0.009) and mpMRI examination (*P* < 0.001) (Table 3). The multivariate models for PCa (AUC = 0.79, *P* = 0.003) and CSPCa (AUC = 0.84, *P* = 0.036) were significantly higher than mpMRI examination and other single parameters in diagnostic accuracy (Fig. 1).

Using the same sensitivity for the multivariate model as the mpMRI examination to detect PCa, the multivariate models would reduce the number of biopsies by 5% compared with mpMRI examination. Using the same specificity for the multivariate model as the mpMRI examination to detect PCa, the multivariate model could increase the number of diagnosed cancers by 4% compared with mpMRI examination.

## Discussion

Prostate biopsy is the gold standard for PCa diagnosis. Despite the safety of this method, it remines controversial as an invasive mean that can come with physical and psychological distress for patients with PSA levels in the gray zone^4^. In our study, we assessed the performance of age, tPSA, fPSA, (f/t)PSA, PSAD, prostate volume, and mpMRI examination in predicting of PCa and CSPCa among patients with tPSA level in the gray zone. The study revealed that log-transformed PSAD, prostate volume, and mpMRI examination were independent predictors for both PCa and CSPCa. Additionally, we developed model based on clinical variables including mpMRI examination. The multivariate model outperformed mpMRI and other single clinical parameters in diagnostic of PCa and CSPCa. Use the multivariate model could reduce the number of prostate biopsies by 5% compared with the use of mpMRI examination.

PSA is one of the important biomarkers for detecting prostate cancer, guiding decisions about prostate biopsy, and offing a way to monitor disease progression^4,15^. Now PCa screening relies primarily on tPSA, fPSA, (f/t)PSA and digital rectal examination (DRE). PSA is a serine protease, which is highly tissue specific, but not cancer specific. The tPSA increases in PCa, as well as in prostatitis and prostatic hyperplasia^16^. The tPSA levels in PCa and benign prostatic hyperplasia overlap, in large part, at a range of 4–10 ng/ml^17^. Our study and other study also showed that the differences of tPSA level between PCa and non-PCa group were not significant among patients with tPSA in the gray zone^18,19^. PSA can be present in free and complexed forms in the serum. The concentration of fPSA is lower in cancer patients than in benign prostate hyperplasia^10^. The (f/t)PSA is helpful to distinguish early PCa from benign prostate hyperplasia. However, the differences of (f/t)PSA ratio were not significant between PCa and non-PCa group in some studies^8^. The inconsistent results of (f/t)PSA among studies may caused by the unstable of fPSA in serum^8,9^. These results may suggest that it is not robust to screen and diagnose PCa in the gray zone based on (f/t)PSA. Our study also showed that tPSA, fPSA, and (f/t)PSA were not significant predictors for CSPCa.

In our study, the prostate volume was smaller among patients with PCa than those with benign prostatic hyperplasia, and larger prostate volume was associated with a lower positive biopsy rate. Previous studies also showed that the PCa detection rate in men decreased with increasing prostate volume^20–22^. The European Randomized Study of Screening for Prostate Cancer (ERSPC) conducted in 2010 and 2012 demonstrated the key role of prostate volume in the prediction of PCa^23,24^. Another clinical parameter, PSAD, was based on the idea that PCa secretes a greater amount of PSA into the blood per unit of prostate volume compared with benign prostate hyperplasia. Several studies have confirmed the importance of PSAD in diagnosis of PCa^25–27^. These results are consistent with our results. Therefore, PSAD offers better guidance in the decision as to whether to conduct prostate biopsy when PSA levels are in the gray zone. In our study, prostate volume and PSAD were independent clinical parameters for predicting of PCa and CSPCa. However, the diagnostic accuracy of PSAD was low in predicting of PCa (AUC = 0.67) and CSPCa (AUC = 0.68).

In recent years, a growing body of literature has validated the clinical utility of mpMRI including T2-weighted imaging(T2WI), diffusion-weighted imaging(DWI) and dynamic contrast-enhanced(DCE) for the detection of CSPCa and to guide clinical decisions^10,11^. In addition, magnetic resonance spectroscopic imaging (MRSI) has demonstrated satisfactory performance in early-stage PCa detection through the analysis of three metabolites (choline, creatine, and citrate)^15,16^. However, the knowledge for the performance of mpMRI including T2WI, WDI, DCE, and MRSI, among patients with tPSA limited in the gray zone is limited. Our study evaluated the diagnostic accuracy of mpMRI in predicting of PCa and CSPCa. For PCa, the diagnostic performance of mpMRI (AUC = 0.69) was similar with prostate volume (AUC = 0.68) and PSAD (AUC = 0.67); for CSPCa, the mpMRI (AUC = 0.79) outperformed prostate volume (AUC = 0.69) and PSAD (AUC = 0.68). These results were in line with the findings that the mpMRI examination was more sensitive in detecting International Society of Urological Pathology (ISUP) grade ≥ 2 PCa than in detecting ISUP grade group 1 PCa^13^. Additionally, we found that only the mpMRI results between CSPCa and non-CSPCa groups were significant, while other clinical parameters including age, tPSA, fPSA, (f/t)PSA, PSAD, and prostate volume, were not helpful in distinct CSPCa from non-CSPCa. A recent meta-analysis showed that the addition of the Prostate Imaging Reporting and Data System (PI-RADS) score increases the sensitivity and specificity of mpMRI for PCa diagnosis^28^. In our stuty, the mpMRI results were divided into three groups according to the reports: “negative”, “equivocal” and “suspicious” for the presence of PCa. The AUC of mpMRI was lower than that of the PI-RADS version 2 (PI-RADS v2) (0.794 for PCa, and 0.855 for CSPCa) among patients with tPSA in the gray zone^29^. These differences further demonstrate that the PI-RADS v2 could be used as a reliable predictor of PCa and CSPCa among patients in the PSA gray zone.

Furthermore, we developed the multivariate models to predict PCa and CSPCa among patients in the PSA gray zone. The models outperformed mpMRI examination and other single clinical parameters for predicting PCa and CSPCa in our study. This indicated that the models could predict of PCa and CSPCa well, which provided a more certain way of predicting PCa, and guiding the clinical decisions. Several study also reported that combining mpMRI examination with other markers, such as PSAD^1^, prostate volume^29^, and the prostate cancer antigen 3 (*PCA3*) gene^30,31^, could improve diagnostic performance and avoid of unnecessary biopsy. Overall, our study will provide basis for establishing the clinical parameter-based diagnostic model of PCa and CSPCa among Chinese population. PCa genomic biomarkers is able to predict the likelihood of an initial positive biopsy; to reduce the number of unnecessary repeat biopsies, and to sub-stratify low-, intermediate-, and high-risk tumors^32^. In the future, the multivariate model combining genomic marker and clinical parameters, should be developed to better identify PCa and avoid unnecessary invasive procedures.

This study was subject to several limitations. First, this study was a single, tertiary-care institution study, and limited by the inherent drawbacks of its retrospective design. Second, the number of CSPCa with gray PSA value was small (n = 28). This may artificially inflate the statistical power of the multivariate analysis. However, this limitation applies to all other similar studies^18,29,33^. Third, we acknowledge that the inclusion of more clinical parameters, for example, DRE results, family history, and genomic marker may have augmented our prediction models and may be considered for future studies. However, the advantage of our study is its simplicity, which could facilitate it implementation in clinical practice.

## Conclusions

Our study demonstrated that prostate volume, PSAD, and mpMRI examination were independent predictors of PCa and CSPCa among patients with tPSA in the gray zone. The multivariate models could be used as an aid to identify PCa and CSPCa among men in the PSA gray zone and reduce unnecessary biopsies without compromising the ability to diagnose PCa and CSPCa. Further prospective validation is required.

## Participants and methods

### Study population

This retrospective study was approved by the review board at out institution. We identified 1227 patients underwent transrectal ultrasound (TRUS)-guided prostate biopsy between May 2014 and September 2018 at our hospital. Of these cases, 242 (19.7%) were patients with tPSA levels in the gray zone. Four cases of stromal sarcoma and three cases of mucinous adenocarcinoma were excluded leaving 235 patients. The enrolled patients were divided into two groups (PCa and non-PCa groups) according to pathological results. Non-PCa was defined by the absence of positive biopsy findings and included cases of benign prostatic hyperplasia, prostatitis, prostatic hyperplasia, and normal prostate tissue with calcification. PCa was defined by prostate adenocarcinoma for any biopsy needle sample. CSPCa was defined by PCa with Gleason score ≥7.

### Clinical parameters collection

The clinical parameters consisted of age at prostate biopsy, serum tPSA and fPSA value, left-right diameter, anteroposterior diameter, and vertical diameter of prostate, and reports of mpMRI examination were extracted form clinical records. Serum tPSA and fPSA measured by immunofluorescence assay before prostate biopsy. Prostate volume was measured by using ultrasonography scanner (BK Medical, Denmark) or 3.0-T MRI system (SIEMENS, Germany) using the exact prolate ellipsoid formula: volume = left-right diameter × anteroposterior diameter × vertical diameter × π/6^34^. All prostate mpMRI examinations were performed using the 3.0-T MRI system. The mpMRI protocol fulfilled the guidelines of the European Society of Urology Radiology, and included T2WI, DWI, DCE perfusion imaging, and MRSI. The prostate mpMRI images were analyzed by two experienced radiologists. The mpMRI results were divided into three groups according to the reports: “negative”, “equivocal” and “suspicious” for the presence of PCa.

### Prostate biopsy and pathological diagnosis

All patients underwent transrectal ultrasound-guided systematic 12-point prostate biopsy^5^. If suspected malignant nodules, additional 1–5 needles were performed in regions with abnormal ultrasound echoes. Biopsy cores were analyzed according to the standards of the ISUP^35^.

### Statistical analyses

We described the profile of age, PSA derivates [tPSA, fPSA, PSAD, (f/t)PSA], prostate volume and mpMRI examination of enrolled patients by pathological diagnosis. The χ^2^ test or Fisher’s exact test was used to analyze categorical data. The Mann-Whitney U test was used to analyze ranked data. Student’s t test or ANOVA was used to analyze continuous data and the Bonferroni method for multiple comparisons was used if a significant difference between groups was noted. Multivariate logistic regression analysis with a stepwise strategy was used to develop models to predict PCa and CSPCa. The area under the ROC curve (AUC) was used to measure the diagnostic accuracy. Differences between the AUCs were compared using the method of DeLong *et al*. Data cleaning and analyses were conducted using R statistical software (Version 3.2.5).

### Ethical statement

The authors are accountable for all aspects of the work in ensuring that questions related to the accuracy or integrity of any part of the work are appropriately investigated and resolved. The study was performed in accordance with the Declaration of Helsinki and all of the participants gave their informed consent.

## References

[1] Moore CM (2017). Reporting Magnetic Resonance Imaging in Men on Active Surveillance for Prostate Cancer: The PRECISE Recommendations-A Report of a European School of Oncology Task Force. European urology.

[2] Ye D, Zhu Y (2015). Epidemiology of prostate cancer in China: an overview and clinical implication. Zhonghua wai ke za zhi [Chinese journal of surgery].

[3] Zhang Yi-Yan, Li Qin, Xin Yi, Lv Wei-Qi (2019). Differentiating Prostate Cancer from Benign Prostatic Hyperplasia Using PSAD Based on Machine Learning: Single-Center Retrospective Study in China. IEEE/ACM Transactions on Computational Biology and Bioinformatics.

[4] Xia J (2013). Effects of screening on radical prostatectomy efficacy: the prostate cancer intervention versus observation trial. Journal of the National Cancer Institute.

[5] *European Association of Urology. EAU guidelines on prostate cancer*, https://uroweb.org/guideline/prostate-cancer/ (2019).

[6] Leyten GHJM (2014). Prospective multicentre evaluation of PCA3 and TMPRSS2-ERG gene fusions as diagnostic and prognostic urinary biomarkers for prostate cancer. European urology.

[7] Yoshida K (1999). Levels of free prostate-specific antigen (PSA) can be selectively measured by heat treatment of serum: free/total-PSA ratios improve detection of prostate carcinoma. Clinica chimica acta; international journal of clinical chemistry.

[8] Bachour DM, Chahin E, Al-Fahoum S (2015). Human Kallikrein-2, Prostate Specific Antigen and Free- Prostate Specific Antigen in Combination to Discriminate Prostate Cancer from Benign Diseases in Syrian Patients. Asian Pacific journal of cancer prevention: APJCP.

[9] Stephan C, Lein M, Jung K, Schnorr D, Loening SA (1997). The influence of prostate volume on the ratio of free to total prostate specific antigen in serum of patients with prostate carcinoma and benign prostate hyperplasia. Cancer.

[10] Huang Y, Li ZZ, Huang YL, Song HJ, Wang YJ (2018). Value of free/total prostate-specific antigen (f/t PSA) ratios for prostate cancer detection in patients with total serum prostate-specific antigen between 4 and 10 ng/mL: A meta-analysis. Medicine.

[11] Porcaro AB (2015). Prostate volume index and chronic inflammation of the prostate type IV with respect to the risk of prostate cancer. Urologia internationalis.

[12] Borofsky S (2018). What Are We Missing? False-Negative Cancers at Multiparametric MR Imaging of the Prostate. Radiology.

[13] Bratan F (2013). Influence of imaging and histological factors on prostate cancer detection and localisation on multiparametric MRI: a prospective study. European radiology.

[14] Van Neste L (2016). Detection of High-grade Prostate Cancer Using a Urinary Molecular Biomarker-Based Risk Score. European urology.

[15] Zhu Y (2016). Effect of body mass index on the performance characteristics of PSA-related markers to detect prostate cancer. Scientific reports.

[16] Catalona WJ (2017). Comparison of Digital Rectal Examination and Serum Prostate Specific Antigen in the Early Detection of Prostate Cancer: Results of a Multicenter Clinical Trial of 6,630 Men. The Journal of urology.

[17] Alonzo DG, Mure AL, Soloway MS (2013). Prostate cancer and the increasing role of active surveillance. Postgraduate medicine.

[18] Zhan Wei-Wei, Liu Jun, Wang Zhi-Qian, Li Min, Zhou Ming-Yang, Yu Yi-Fei (2020). Establishment of two new predictive models for prostate cancer to determine whether to require prostate biopsy when the PSA level is in the diagnostic gray zone (4–10 ng ml−1). Asian Journal of Andrology.

[19] Erdogan Abdullah, Polat Salih, Keskin Ercument, Turan Abdullah (2019). Is prostate volume better than PSA density and free/total PSA ratio in predicting prostate cancer in patients with PSA 2.5–10 ng/mL and 10.1–30 ng/mL?. The Aging Male.

[20] Karakiewicz PI (1997). Outcome of sextant biopsy according to gland volume. Urology.

[21] Wu YS (2014). The influence of prostate volume on cancer detection in the Chinese population. Asian journal of andrology.

[22] Al-Khalil S (2016). Interactions between benign prostatic hyperplasia (BPH) and prostate cancer in large prostates: a retrospective data review. International urology and nephrology.

[23] Cavadas V (2010). Prostate cancer prevention trial and European randomized study of screening for prostate cancer risk calculators: a performance comparison in a contemporary screened cohort. European urology.

[24] Roobol MJ (2012). Importance of prostate volume in the European Randomised Study of Screening for Prostate Cancer (ERSPC) risk calculators: results from the prostate biopsy collaborative group. World journal of urology.

[25] Froehner M, Buck LM, Koch R, Hakenberg OW, Wirth MP (2009). Derivatives of prostate-specific antigen as predictors of incidental prostate cancer. BJU international.

[26] Lin YR (2015). PSA density improves the rate of prostate cancer detection in Chinese men with a PSA between 2.5–10.0 ng ml (-1) and 10.1–20.0 ng ml (-1): a multicenter study. Asian journal of andrology.

[27] Sozen S (2005). Complexed prostate specific antigen density is better than the other PSA derivatives for detection of prostate cancer in men with total PSA between 2.5 and 20 ng/ml: results of a prospective multicenter study. European urology.

[28] Zhang L (2017). A meta-analysis of use of Prostate Imaging Reporting and Data System Version 2 (PI-RADS V2) with multiparametric MR imaging for the detection of prostate cancer. European radiology.

[29] Liu C (2018). Using the prostate imaging reporting and data system version 2 (PI-RIDS v2) to detect prostate cancer can prevent unnecessary biopsies and invasive treatment. Asian journal of andrology.

[30] Lanz C (2016). Gleason Score Determination with Transrectal Ultrasound-Magnetic Resonance Imaging Fusion Guided Prostate Biopsies–Are We Gaining in Accuracy?. The Journal of urology.

[31] Kaufmann S (2016). Prostate cancer gene 3 (PCA3) is of additional predictive value in patients with PI-RADS grade III (intermediate) lesions in the MR-guided re-biopsy setting for prostate cancer. World journal of urology.

[32] Cucchiara V (2018). Genomic Markers in Prostate Cancer Decision Making. European urology.

[33] Deniffel Dominik, Zhang Yucheng, Salinas Emmanuel, Satkunasivam Raj, Khalvati Farzad, Haider Masoom A. (2020). Reducing Unnecessary Prostate Multiparametric Magnetic Resonance Imaging by Using Clinical Parameters to Predict Negative and Indeterminate Findings. Journal of Urology.

[34] Christie DRH, Sharpley CF (2019). How Accurately Can Prostate Gland Imaging Measure the Prostate Gland Volume? Results of a Systematic Review. Prostatic Dis.

[35] Egevad L (2016). International Society of Urological Pathology (ISUP) Grading of Prostate Cancer. The American journal of surgical pathology.

